# Effects of Anesthetic Agents on Brain Blood Oxygenation Level Revealed with Ultra-High Field MRI

**DOI:** 10.1371/journal.pone.0032645

**Published:** 2012-03-12

**Authors:** Luisa Ciobanu, Olivier Reynaud, Lynn Uhrig, Béchir Jarraya, Denis Le Bihan

**Affiliations:** 1 NeuroSpin, Commissariat à l'Energie Atomique et aux Energies Alternatives, Gif-sur-Yvette, France; 2 Equipe Avenir INSERM Bettencourt Schueller, NeuroSpin, Institut Fédératif de Recherche n°49, Gif-sur-Yvette, France; 3 Unité de Neurochirurgie Fonctionnelle, Henri-Mondor Hospital, Créteil, France; Kaohsiung Chang Gung Memorial Hospital, Taiwan

## Abstract

During general anesthesia it is crucial to control systemic hemodynamics and oxygenation levels. However, anesthetic agents can affect cerebral hemodynamics and metabolism in a drug-dependent manner, while systemic hemodynamics is stable. Brain-wide monitoring of this effect remains highly challenging. Because T_2_*-weighted imaging at ultra-high magnetic field strengths benefits from a dramatic increase in contrast to noise ratio, we hypothesized that it could monitor anesthesia effects on brain blood oxygenation. We scanned rat brains at 7T and 17.2T under general anesthesia using different anesthetics (isoflurane, ketamine-xylazine, medetomidine). We showed that the brain/vessels contrast in T_2_*-weighted images at 17.2T varied directly according to the applied pharmacological anesthetic agent, a phenomenon that was visible, but to a much smaller extent at 7T. This variation is in agreement with the mechanism of action of these agents. These data demonstrate that preclinical ultra-high field MRI can monitor the effects of a given drug on brain blood oxygenation level in the absence of systemic blood oxygenation changes and of any neural stimulation.

## Introduction

Anesthetic drugs are widely used in clinical surgery, intensive care units and preclinical research. Although it is well established that anesthetic agents influence the cerebral blood flow (CBF) [1] and cerebral metabolism [2], the data reported are often conflicting. For example, the study of ketamine effects on CBF and cerebral metabolic rate of oxygen (CMRO_2_) found contradictory results in patients [3], [4], and in different animal models [5], [6]. Effects might also differ locally: ketamine caused simultaneous increase in CBF and glucose utilisation only in specific limbic structures [7]. Compared to the awake state, ketamine-induced anesthesia in rats caused no change in CBF and CMRO_2_ except in the anterior cortex which showed a small increase in CMRO_2_ [8]. Lei et al. [9] showed that xylazine generated a strongly region-dependent reduction in CBF, with the largest reductions in the hypothalamus and septum and the least reduction in the caudate putamen. The same authors [9] showed that ketamine induced no significant increase in the CBF in the rat, supporting the idea that there are no major changes in CMRO_2_ in the cortex of ketamine anesthetized rats. Thus, the conflicting findings about ketamine consequences may be related to the dosage, the ventilation methods, the species differences and the methodology applied to measure cerebral blood flow and metabolism. Because a deep understanding of the effects of anesthetic drugs on cerebral hemodynamics and metabolism is critical both for the clinical and preclinical use as well as for translational research, there is a need for new sensitive and specific measurement methods.

MRI has been shown to be sensitive to blood oxygenation effects, especially when using high magnetic fields, due to the paramagnetic properties of deoxyhemoglobin contained in circulating erythrocytes [10]–[12]. Deoxyhemoglobin introduces magnetic field inhomogeneities, while oxyhemoglobin, being diamagnetic, has a negligible effect. As a result, a decrease in deoxyhemoglobin leads to an increase in signal intensity in magnetic resonance images sensitized to the apparent transverse relaxation time (T_2_*), e.g. gradient echo images. Thus we hypothesized that a substantial increase in the MR field would sensitize the signal to a degree that would enable T_2_* signal to directly monitor brain blood oxygenation level.

Here we show that different anesthetic agents (isoflurane, ketamine-xylazine, medetomidine) spontaneously induce different local contrast changes in magnitude gradient echo images acquired at ultra-high magnetic field (17.2T) and high magnetic field (7T) *in-vivo* in rat brains. Such changes are directly related to effects of the drugs on the microvasculature and are readily quantifiable. Our results suggest that ultra-high field (UHF) MRI is a good candidate to assess *in vivo* and in real-time the effects of various drugs on brain blood oxygenation level.

## Results

### Physiologic measurements

There were no significant differences in arterial blood-gas values between different types of anesthesia protocols: PaCO_2_ was measured in a range of 41–46 mmHg, PaO_2_ was >200 mmHg and pH was maintained in a 7.30–7.45 range. During all the MR experiments the ventilation parameters were adjusted to maintain constant exhaled CO_2_. The mean arterial systolic blood pressure was 80.0±3.6 mmHg under isoflurane, 69.9±3.6 mmHg under ketamine-xylazine and 87.0±9.3 mmHg under medetomidine.

### Effect of the magnetic field strength on T_2_* contrast

**Figure 1:**
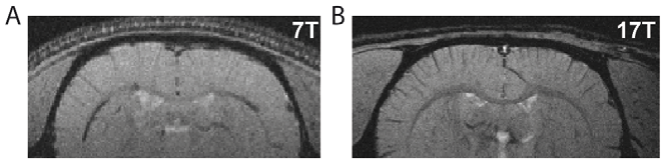
Effect of the magnetic field strength on T_2_* contrast in rat brain images acquired *in vivo* under ketamine-xylazine anesthesia. Representative FLASH images, coronal sections, acquired at 7T (A) and 17.2T (B). Image resolution: 100 µm×100 µm×250 µm and 80 µm×80 µm×200 µm for 7T and 17.2T, respectively.

Figure 1 shows coronal magnitude images obtained, under the same anesthesia protocol (ketamine-xylazine), at two different magnetic field strengths, 7T (Figure 1A) and 17.2T (Figure 1B). Vein-parenchyma contrast was higher at 17.2T compared to 7T. Specifically, for the rats imaged in Figure 1 the number of hypointense pixels counted at 7T and 17.2T were 197 and 516, respectively (8 slices, total number of pixels considered 20 000). Similar results were obtained under medetomidine anesthesia: we counted 158 hypointense pixels at 7T and 419 at 17.2T.

### Anesthesia effect on magnitude T_2_* contrast

#### Isoflurane vs ketamine-xylazine

**Figure 2:**
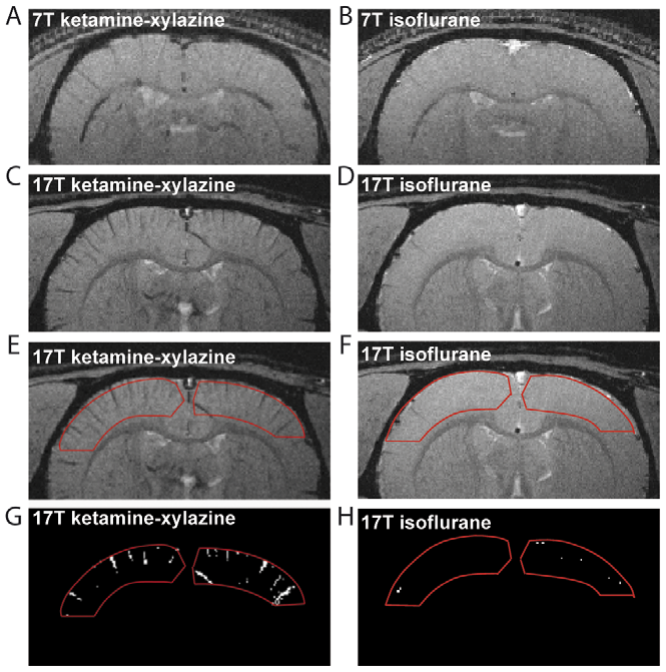
Effect of anesthetic agents on T_2_* contrast in rat brain images at 7T and 17.2T: ketamine-xylazine vs isoflurane. Images are coronal sections at 7T (A, B) and 17.2T (C, D, E, F) acquired *in vivo* under general anesthesia using ketamine-xylazine (A, C, E) and isoflurane (B, D, F). The red ROIs (E, F) show the regions used to calculate the number of pixels corresponding to visible blood vessels at 17.2T. The pixels below the 75% intensity threshold (see text) corresponding to these ROIs are clearly visible (G, H).

Coronal magnitude T_2_* images obtained under the two different types of anesthesia (isoflurane and ketamine-xylazine) at different field strengths (7T and 17.2T) are shown in Figure 2. We notice a slight change in contrast at 7T after the injection of ketamine-xylazine (Figures 2A and 2B). On the other hand, at 17.2T the change in contrast is substantial. The vein-parenchyma contrast under ketamine-xylazine anesthesia is higher compared to that under isoflurane anesthesia. Being oriented parallel to the slice plane, the blood vessels appear as dark lines; as it is clearly seen in Figure 2C. The analysis performed on the 17.2T magnitude data revealed a striking contrast increase. Specifically, in analyzing the signal intensity in the cortex we found 5.4 times (average over 16 slices, in 3 animals) more pixels corresponding to blood vessels, when using ketamine-xylazine anesthesia versus isoflurane anesthesia. The results obtained for each animal are shown in Table 1. Figures 2G and 2H show typical examples of pixels counted in our analysis for the two anesthesia conditions, corresponding to the ROIs displayed in Figures 2E and 2F, respectively.

**Table 1:** Ratio of the number of hypointense pixels counted for different anesthesia conditions (ketamine-xylazine/isoflurane and medetomidine/isoflurane) at 17.2T.

Anesthetic	Rat #	Hypointense pixels under isoflurane	Ratio of hypointense pixels
Ketamine-xylazine/isoflurane	1	225	4.1
	2	238	4.4
	3	180	7.7
Medetomidine/isoflurane	4	196	7.2
	5	134	4.0
	6	241	3.4

#### Isoflurane vs medetomidine

**Figure 3:**
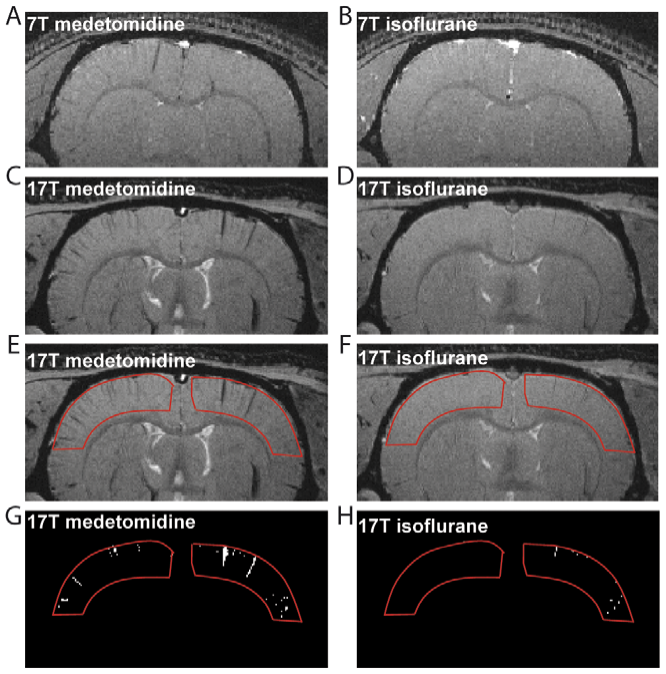
Effect of anesthetic agents on T_2_* contrast in rat brain images at 7T and 17.2T: medetomidine vs isoflurane. Images are coronal sections at 7T (A, B) and 17.2T (C, D, E, F) acquired *in vivo* under general anesthesia using ketamine-xylazine (A, C, E) and isoflurane (B, D, F). The red ROIs (E, F) show the regions used to calculate the number of pixels corresponding to visible blood vessels at 17.2T. The pixels below the 75% intensity threshold (see text) corresponding to these ROIs are clearly visible (G, H).

To ascertain which anesthetic agent is mainly responsible for the difference in contrast described above we performed a second set of experiments in which we compared isoflurane with medetomidine. Figure 3 shows the images acquired, at 7T and 17.2T, under the two conditions: T_2_* contrast obtained under medetomidine is very similar to the contrast obtained under ketamine-xylazine (Figure 3C). Image analysis showed a 4.8 times increase in the number of the blood vessels counted under medetomidine compared to that under isoflurane (average over 16 slices in 3 animals). The images analysis was performed as for the isoflurane ketamine-xylazine comparison with typical examples of pixels counted shown in Figures 3G and 3H, corresponding to the ROI displayed in Figures 2E and 2F, respectively. Table 1 summarizes the results.

**Figure 4:**
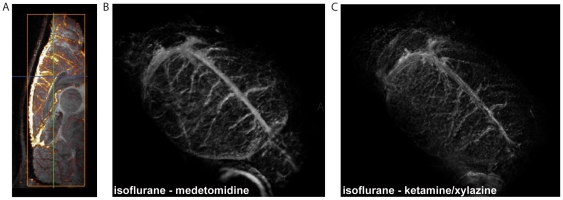
Difference images obtained by subtracting images acquired under two anesthesia conditions at 17.2T. (A) isoflurane - medetomidine, sagittal plane (the image difference is superimposed on the image acquired under medetomidine) (B) isoflurane – medetomidine 3D rendering (C) isoflurane – ketamine-xylazine 3D rendering Acquisition parameters: resolution 120 µm×120 µm×120 µm, flip angle α = 40°, TR/TE = 200/8 ms.

We limited our image analysis to cortical regions; however, blood vessels are also visible in sub-cortical regions, including the thalamus and hippocampus (both visible in Figure 3). 3D acquisitions are also possible as illustrated in Figure 4, which displays the difference between images acquired under isoflurane and medetomidine anesthesia overlaid on the medetomidine image (Figure 4A) and the 3D renderings of the difference images between isoflurane ketamine-xylazine and isoflurane medetomidine, in Figures 4B and 4C, respectively. Veins and their ramifications are clearly visible on the brain surface.

## Discussion

In this study we establish that the brain/vessels contrast in T_2_*-weighted images at UHF manifestly depends on the anesthetic agent used. Stemming from magnetic susceptibility differences between the blood in the vessels and the surrounding tissue, this phenomenon is visible to a much smaller extent at lower field strengths (7T). To our knowledge this is the first experimental observation of this effect. The different anesthetic agents, at the concentrations that we used, are known to induce ‘loss of consciousness’ in rodents. Furthermore, clinical observation and physiology monitoring indicated a stable general anesthesia in all animals. Could the anesthetic agents act as contrast agents? Because anesthetic agents induce profound changes in general and local brain hemodynamics and metabolism, a plausible explanation for these results is the difference in the deoxyhemoglobin brain vascular content induced by the anesthesia. This difference is caused by changes in different physiological parameters (CBF, CMRO_2_, PtO_2_, vasodilatation) [13]–[22] generated by the three anesthetics used in this study (isoflurane, ketamine-xylazine, medetomidine). The modifications observed in the vessel-tissue contrast in the UHF T_2_* images are induced by changes in blood oxygen level produced by an altered metabolic load or altered CBF. Under normoxic conditions arterial blood is fully oxygenated and does not contribute to blood oxygenation level dependent (BOLD) contrast, while venous blood vessels containing de-oxygenated blood show as hypointense regions in the MR images. BOLD image contrast is enhanced at high magnetic fields, as we see on the images, with the hypointense lines better visible at 17.2T than at 7T. BOLD contrast can be used to monitor non-invasively the blood oxygenation levels of the brain in response to central nervous system drugs that affect basal metabolism or CBF, like the anesthesia agents used in this paper. Isoflurane resulted in a low contrast between cortex and venous blood vessels as seen in the 17.2T images, likely due to CBF increase, cerebral glucose uptake decrease and decrease of deoxygenated blood in the venous blood vessels. Ketamine-xylazine and medetomidine resulted in a high contrast between cortex and venous blood vessels, probably because of CBF decrease and cerebral glucose utilization increases in some limbic structures, leading to an increase of deoxygenated blood in the venous blood vessels.

Further investigations of this dependence are needed to fully understand the relationship between the anesthetic agent used and the vessel-tissue contrast observed. The majority of animal fMRI studies (rodents, non-human primates) are performed under general anesthesia in order to reduce motion artifacts and to minimize the stress [23], [24]. Not only do the anesthetic agents influence the brain activity [25], [26] but also modulate the signal intensity in T_2_*-weighted images in UHF as shown in this manuscript. It is therefore imperative to know which anesthetic agent is most appropriate when performing fMRI studies. Knowing the influence of different anesthetic agents on T_2_* will help the optimization of scanning parameters, especially the echo time (TE), given the strong dependence of the functional activation pattern on the TE/T_2_* relationship [27].

MRI at UHF allows an increase in resolution and image contrast. The increase in contrast to noise ratio is particularly important for T_2_*-weighted imaging, which is sensitive to susceptibility effects caused by a variety of sources, including deoxyhemoglobin concentration, iron deposits, and tissue microstructure. In the human brain, gradient echo techniques at UHF produce high resolution, high contrast images [28] and are able to delineate cortical vascular anatomy and resolve micro-vessels with diameters down to 100 µm both *in vitro* [29] and *in vivo* [30], [31]. The visualization of small venous structures is very useful for studying neurological diseases in relevant preclinical animal models. A few studies demonstrated the non-invasive detection of rodent brain vasculature in magnitude T_2_*-weighted images at high magnetic fields [10], [12]. More recent studies report high resolution imaging of brain microvasculature in rodents using susceptibility weighted imaging [32] or phase imaging [33]. As shown in this manuscript, due to the extreme sensitivity to brain blood oxygenation level changes induced by anesthetic agents, UHF MR studies have the potential to screen future anesthesia drugs for their action on cerebral blood oxygentaion. More generally, UHF may also play a role during preclinical screening of new pharmacological agents for their effect on brain oxygenation on a local basis.

## Materials and Methods

### Animals

All animal studies were conducted in accordance with the European convention for animal care and the NIH's Guide for the Care and Use of Laboratory Animals. This study has been approved by the Comité d'EThique en Expérimentation Animale Commissariat à l'Energie Atomique et aux énergies alternatives Direction des Sciences du Vivant Ile de France (CETEA CEA DSV IdF) under protocol ID 10_032. Sprague-Dawley male rats with weights between 275 g and 300 g (N = 10) were obtained from Janvier (Saint Isle, France).

### General anesthesia

During general anesthesia, animals were mechanically ventilated (Bioseb, Vitrolles, France) and received a mixture of air/oxygen (FiO_2_ = 0.33). All available physiological parameters (blood pressure, respiration rate, expired CO_2_, O_2_ saturation, temperature) were monitored and kept constant throughout the experiment to ensure normocapnic and normoxic conditions (for the experiments performed on the 17.2T the O_2_ saturation was not monitored due to the incompatibility of the monitoring system with the strong magnetic field). Arterial blood gases (pH, PaO_2_, PaCO_2_) were sampled a first time after the intubation but before the beginning of the acquisition, and the second time immediately at the end of the MRI measurement for each anesthesia condition and analyzed using a blood gas analyzer (Radiometer Copenhagen).

One group of six animals was imaged on the 17.2T system. Out of these, three animals were imaged under isoflurane and medetomidine anesthesia while the other three were imaged under isoflurane and ketamine-xylazine anesthesia.

Initially, the animals were anesthetized and maintained under isoflurane (2% inspired isoflurane) during which time a series of gradient echo images was acquired. Next the animals were injected with a bolus of medetomidine (0.3 mg/kg, i. v.) or of ketamine-xylazine (100/10 mg/kg, i.p.) and the isoflurane was discontinued. A new set of gradient echo images was acquired 30 minutes after isoflurane was turned off. For comparison four animals were imaged on the 7T, two under each of the two anesthesia conditions. For two of these animals (one in each anesthesia group) we installed a femoral catheter and invasively monitored the blood pressure (SAII, Stony Brook, USA).

### MRI acquisition

The UHF MR experiments were performed on a horizontal bore, 17.2T BioSpec (Bruker BioSpin, Etlingen, Germany) imaging system equipped with a maximum gradient strength of 1000 mT/m. A 3 cm diameter surface coil (Bruker BioSpin, Etlingen, Germany) was used for transmission and reception. On the 7T (PharmaScan, Bruker Biospin, Etlingen, Germany) the experiments were performed using a home build 2.5 cm diameter surface coil. Following scout scans and magnetic field homogeneity optimization (FASTMAP), coronal T_2_^*^ gradient echo images were acquired. The 2D acquisition parameters were optimized for the two different field strengths as follows: 17.2T: in-plane resolution 80 µm, FOV = 25.6 mm×25.6 mm (matrix size 320×320), flip angle α = 45°, TR/TE = 350/8 ms, thk = 0.2 mm, N_slice = 16, NEX = 14, 7T: in-plane resolution 100 µm, FOV = 25.6 mm×25.6 mm (matrix size 256×256), TR/TE = 300/12 ms, thk = 0.25 mm, NEX = 20. For the 3D images acquired on the 17.2T system we used the following parameters: resolution 120 µm×120 µm×120 µm, flip angle α = 45°, TR/TE = 200/8 ms.

### Data processing

The 2D MR images were analyzed in Matlab 1.2 (MathWorks, Natick, Massachusetts). Regions of interest (ROIs) were selected in areas of high blood vessel density (cortical regions). For each ROI we extracted the average signal intensity and then counted the number of pixels with intensities smaller than 75% of this average. The contrast to noise ratio (CNR) between tissue and vascular pixels can be calculated as a function of this 75% threshold (τ) and the signal to noise ratio (SNR) of the images using the relationship: CNR = (1−τ)×SNR. Given the SNR of 28±1.7 for our images (measured in regions without prominent blood vessels) we found CNR = 7, which exceeds the value required for reasonable discrimination [34]. In choosing the ROIs, the very bright pixels corresponding to edge artefacts were excluded.

The 3D images were processed using Amira software (Amira 5.3.3, TGS, San Diego, CA). The two datasets obtained under different anesthesia were realigned and the difference image was generated on a pixel-by-pixel basis and visualized with Amira 3D rendering.
